# Survey of viral populations within Lake Michigan nearshore waters at four Chicago area beaches

**DOI:** 10.1016/j.dib.2015.08.001

**Published:** 2015-08-13

**Authors:** Emily Sible, Alexandria Cooper, Kema Malki, Katherine Bruder, Siobhan C. Watkins, Yuriy Fofanov, Catherine Putonti

**Affiliations:** a Department of Biology, Loyola University Chicago, 1032 W Sheridan Rd, Chicago, IL 60660, USA; b Department of Pharmacology and Toxicology, University of Texas Medical Branch, 5.112 BSB, Galveston, TX 77555, USA; c Bioinformatics Program, Loyola University Chicago, 1032 W Sheridan Rd, Chicago, IL 60660, USA; d Department of Computer Science, Loyola University Chicago, 820 N Michigan Ave, Chicago, IL 60611, USA

**Keywords:** Viral community, Metagenomics, Freshwater, Lake Michigan

## Abstract

In comparison to the oceans, freshwater environments represent a more diverse community of microorganisms, exhibiting comparatively high levels of variability both temporally and spatially Maranger and Bird, Microb. Ecol. 31 (1996) 141–151. This level of variability is likely to extend to the world of viruses as well, in particular bacteria-infecting viruses (bacteriophages). Phages are known to influence bacterial diversity, and therefore key processes, in environmental niches across the globe Clokie et al., Bacteriophage 1 (2011) 31–45; Jacquet et al., Adv. Ocean Limn. 1 (2010) 97–141; Wilhelm and Suttle, Bioscience 49 (1999) 781–788; Bratback et al., Microb. Ecol. 28 (1994) 209–221. Despite their prevalence and likely critical role in freshwater environments, very few viral species have been characterized. Metagenomic approaches, however, have allowed for a glimpse into phage diversity. We collected surface water samples from four Chicago area beaches – Gillson Park, Montrose Beach, 57th Street Beach, and Calumet Beach – every two weeks from May 13 through August 5, 2014. Sampling was conducted with four biological replicates for each sampling date and location, resulting in 112 samples. DNA isolated from each of the individual samples for a given collection date/location was pooled together, with one exception – Calumet Beach on August 5, 2014 – in which each biological replicate was sequenced individually. Raw sequence data is available via NCBI’s SRA database (part of BioProject PRJNA248239).

**Specifications Table** [please fill in right-hand column of the table below]

**Table:** 

Subject area	*Biology*
More specific subject area	*Viral metagenomics*
Type of data	*Text files: sequences*
How data was acquired	*Illumina MiSeq Desktop Sequencer*
Data format	*Raw*
Experimental factors	*DNA extracted from virus-like particles captured using 0.10* μm *filtration through tangential flow filtration system.*
Experimental features	*Genomic DNA was fragmented and then sequenced using the MiSeq Reagent Kit v2 (500-cycles) kit for the Illumina MiSeq platform.*
Data source location	Chicago, IL, USA: Montrose Beach (41°58′0.71″N, 87°38′13.35″W), 57th Street Beach (41°47′25.54″N, 87°34′41.25″W), and Calumet Beach (41°43′8.18″N, 87°31′32.51″W); Wilmette, IL, USA: Gillson Park (42°4′45.10″N, 87°40′59.10″W).
Data accessibility	*Raw data is available through NCBI’s BioSample database by this link. BioSample IDs include:* SAMN03407346, SAMN03408283, SAMN03435782, SAMN03435784, SAMN03435787, SAMN03435791, SAMN03435794, SAMN03435808, SAMN03435814, SAMN03435817, SAMN03435819, SAMN03435823, SAMN03435829, SAMN03435836, SAMN03435838, SAMN03435841, SAMN03436866, SAMN03436867, SAMN03436868, SAMN03436869, SAMN03436870, SAMN03436871, SAMN03436872, SAMN03436873, SAMN03436874, SAMN03436875, SAMN03436876, and SAMN03436877.

**Value of the data**

• Despite their ubiquity and importance in maintaining microbial communities [1–5], very few bacteriophage species have been fully characterized, largely due to the difficulty in isolating and propagating phage within the laboratory setting. Nevertheless, metagenomics provides a peek into the functionalities present within phage communities.
• Little is known about the viral species within the freshwaters of the Great Lakes.
• Genomic information produced here provides a baseline which can aid future efforts in determination of the viral diversity present in Lake Michigan.

## 1 Experimental Design, Materials and Methods

### 1.1 Sample collection

Four Chicago area beaches were selected as study sites: Gillson Park (42°4′45.10″N, 87°40′59.10″W), Montrose Beach (41°58′0.71″N, 87°38′13.35″W), 57th Street Beach (41°47′25.54″N, 87°34′41.25″W), and Calumet Beach (41°43′8.18″N, 87°31′32.51″W). All four are recreational swimming areas. The Montrose Beach sampling site is bordered to the north by the Montrose Beach dog park and to the south by the Montrose Harbor Marina. 57th Street Beach and Calumet Beach are used solely for swimming. Gillson Park is located north of Chicago in Wilmette, IL; this beach is recreational and adjacent to the north of Wilmette Harbor. Gillson Park and Calumet Beach are adjacent to locks controlling the movement of water between the North Shore Channel and Calumet River, respectively, and Lake Michigan. (No specific permits or permissions were required for the water samples collected from the Chicago beaches; a permit was obtained for Gillson Park in accordance with the Wilmette Park District.) Each site was sampled with four replicates every two weeks over the three month period—May 13 through August 5, 2014; seven samples (with replicates) were collected for each site. Water was taken from the surface at a distance from the shore such that the water level was approximately knee-deep (~0.5 m deep). Each sample (4L), including each biological replicate, was collected within a 5 m area.

### 1.2 Viral isolation

Isolation of virus-like particles was conducted through successive filtration. The water was first filtered through sterile 0.45 μm bottle-top cellulose acetate membrane filters (Corning Inc, Corning, NY) to remove plant matter, sand, debris, and eukaryotic cells. The filtrate was next passed through a 0.22 μm polyethersulfone membrane filter (MO BIO Laboratories, Carlsbad, CA) to remove bacterial cells. The remaining filtrate was filtered once again, this time through a 0.10 μm polypropylene filter (EMD Millipore Corp, Billerica, MA) using the Labscale™ tangential flow filtration (TFF) system (EMD Millipore Corp, Billerica, MA). Filtration and particle concentration using the TFF was performed according to the manufacturer’s instructions. Each 4L sample was processed individually. The filtrate (~5 ml in total per sample) was then stored at −20 °C until extraction.

### 1.3 DNA extraction

DNA was extracted from all samples using the MO BIO Laboratories UltraClean^®^ DNA Isolation Kit (Carlsbad, CA). The protocol recommended by the manufacturer was followed with the exception of an additional heat treatment at 70 °C for 20 min prior to initial vortexing. Concentrations were verified using the NanoDrop (ThermoScientific, Waltham, MA). To test viral extractions for putative bacterial contamination, each DNA sample was assessed using the 16S 63F/1087R primer pair following standard PCR protocols [6]. Positive (*Escherichia coli* C DNA) and negative (nuclease-free water) were used as controls. None of the extracted DNA samples produced 16S amplicons, suggesting that the DNA isolated was predominately viral. DNA was stored at −20 °C until sequencing.

### 1.4 Library preparation and sequencing

Library construction and sequencing was conducted at the University of Texas Medical Branch (Galveston, TX). DNA was fragmented using NEBNext^®^ Fragmentase (New England Biolabs, Ipswich, MA) into the size of 300 to 400 bp. DNA isolated from each of the individual samples for a given collection date/location was pooled together, with the exception of the Calumet Beach samples from August 5, 2014. Libraries were prepared using the NEBNext^®^ Ultra™ DNA Library Prep kit (New England Biolabs, Ipswich, MA) for Illumina. The samples (27 pooled and 4 individual replicates) were next individually barcoded and sequenced using the Illumina MiSeq platform via the MiSeq Reagent Kit v2 (500 cycle), producing paired-end reads each 250 nucleotides in length.

### 1.5 Sequence demultiplexing

Demultiplexing of the sequence data was automated by the Illumina sequencer’s CASAVA package.
